# Doctor Retention in a COVID-World: An Opportunity to Reconfigure the Health Workforce, or "Plus ça change plus c’est la meme chose"? A Response to the Recent Commentaries

**DOI:** 10.34172/ijhpm.2021.60

**Published:** 2021-06-23

**Authors:** Ruairí Brugha

**Affiliations:** Department of Public Health and Epidemiology, Royal College of Surgeons in Ireland, Dublin, Ireland.

## Introduction

 Let me start by thanking the *International Journal of Health Policy and Management* (IJHPM) and the authors for the seven informative commentaries on Brugha and colleagues’ “*Doctor Retention: A Cross-sectional Study of How Ireland Has Been Losing the Battle.”*^1^ The commentary authors most familiar with the Irish medical workforce, through research^2^and strategy development roles,^3^ are correct in concluding that the research article generated few new insights for Irish readers. The commentaries identified the rich body of research, especially qualitative studies, that has emerged in the last 10 years from a country that ranks unusually high among high income countries for outward emigration of its medical graduates; and has relied too much on international recruitment and inward flows of doctors from mainly low- and middle-income countries (LMICs) to replace them.

 The research article clarified the migration choices of non-consultant hospital doctors (NCHDs or early career doctors) in Ireland; and measured the numbers and described the characteristics of those who were planning to leave, temporarily or permanently. Such information is essential to the design and targeting of policy responses. Large-scale emigration of Irish doctors, which was reported in the 1960s,^1,2^ has deep roots. Those able and ambitious to make a better life elsewhere have been emigrating from Ireland for several centuries, increasingly since the Great Famine of the 1840s; and migration has characterised *homo sapiens *for over 100 000 years. Young doctors especially are pulled towards the opportunities of life experiences and comparatively better training in other countries; and pushed by negative experiences while working and training in their country of graduation.^1-3^ Similar migration patterns and motivations are common among doctors who leave LMICs for high-income countries, with the critical difference that high income countries have the resources and should be able to retain the doctors they train.

## Deepening the Analysis

 Rather than reprise the findings, which the commentaries have already generously done, this response looks to deepen the analysis of underlying systems and policy factors, which were insufficiently explored in the research article, despite IJHPM’s generous word count. As the title implies, the response asks if the coronavirus disease 2019 (COVID-19) pandemic, out of which better-led wealthier countries are emerging, will result in the transformation needed in how countries configure their national health workforces; and in how they value, train and treat the women and men who are the lifeblood of their health services. A deeper analysis of one country’s (Ireland’s) health system will, I believe, be more useful to readers than aspirational principles that risk becoming platitudes around the importance of valuing the global good that is the health workforce.

 Offiah et al have provided an explanatory background to Irish doctor emigration,^3^ much of which we covered in a national policy dialogue on doctor emigration in November 2017^4^; and they have detailed progress made and not yet made, signposting further changes needed to retain Ireland’s NCHDs. Their commentary helps to situate NCHDs’ unsatisfactory working conditions, lack of supervision and mentoring, and their comparatively poor training experiences in the context of the part-successes and part-failures of the measures contained in the national strategy for tackling these longstanding problems. What can also be inferred from the work-related stress and resulting bullying experienced by frontline health professionals – involving trainees, consultants and nurses^5,6^ – is that these problems stem to a great extent from Ireland’s failure to achieve an adequately staffed consultant-delivered, medical workforce model. This, correctly, is their starting point.^3^

 One of the main reasons why Irish doctors – NCHDs and also consultants – migrate to Anglophone countries such as Australia is because there they experience higher ratios of trainers to trainees, 2:1 and greater, compared with 1:2 ratios and worse in Ireland.^7^ Offiah and colleagues’ analysis could go further in that one objective of Ireland’s new national 10-year plan to achieve universal healthcare is to replace with a single tier the two-tier public-private system, where many consultants are currently employed on mixed public private contracts.^8^ These allow them to conduct parallel private practices in adjoining or offsite private hospitals, undermining their supervision and training responsibilities to NCHDs, who take on much of the public practice work that over-worked consultants should be doing. Not all policy obstacles and failures, however, can be placed at the foot of Irish policy-makers and the medical profession. Year-on-year increases in NCHD training positions, essential to providing long-term career paths and permanent careers for doctors in Ireland, have been swamped by a 4-to 5-fold greater increase in non-training posts.^3,4^ Compliance with the European Working Time Directive has forced the State to recruit doctors, almost all of whom are recruited internationally, to staff smaller hospitals that cannot fulfil training criteria, but are currently delivering 24-hour, 7-day acute care.^3,4^

 Deepening the health systems analysis points to an urban: rural divide, common to many countries, which in Ireland’s highly pluralistic democracy is characterised by the political imperative of keeping smaller hospitals open.^3,4^ These provide acute care to small numbers of patients who present to small, sometimes unfit-for-purpose Accident and Emergency departments. This pattern of care persists despite longstanding concerns around overstretched consultant cover and questionable quality of acute care in these hospitals. Such challenges are not unique to Ireland, although its multi-seat political constituency system often elects independent members of parliament whose main political plank is to keep the county hospital open and fully functional. So, what are the possible responses?

 For decades, politicians have known that many small peripheral hospitals need to be converted to facilities for chronic care and day surgery, while giving communities better access to larger hospitals with an adequate complement of consultants and trainees who can provide comprehensive, high quality services. Reconfiguring the medical workforce, by training and employing more generalist hospital consultants, more suited to working in large regional than in national super-specialist hospitals, goes hand-in-hand with hospital restructuring. Several decades of reports have informed policy-makers on the need to address these root causes of doctor emigration, which were largely ignored in Ireland’s 2015 retention strategy.^9^ Since March 2020, the COVID-19 pandemic has demonstrated how an effective health system underpins all dimensions of society. Ireland, which had the lowest proportion of intensive care unit beds in the European Union (EU), escaped some of the worst impacts on hospitals due to an effective public-health led strategy, combined with long periods of stringent lockdowns. This paper ends with the question: will Ireland’s COVID-19 ‘near-miss’ trigger the political will and prioritisation of resources to achieve the goal of consultant-delivered hospital care, in hospitals that are fit for training and can thereby help retain its NCHDs?

## Responses and Solutions

 Moving from the particular (Ireland) to the general, Chevillard’s commentary^10^ helpfully applied the labour market framework of Sousa et al,^11^ which categorises doctor retention policies as addressing three sets of factors: health worker production, inflows and outflows, and maldistribution. In practice, many of the policy levers address two and sometimes all three categories. Some countries use bonding or mandatory deployment of recent graduates to practice for a period in rural areas. This is a part-fix for the maldistribution of doctors, but is less effective at preventing their emigration. Also, the trade-offs and tension between geographical access and quality of care have deep roots, as seen above in Ireland. The most effective measures for addressing inflows and outflows are those that work with, rather than against, the forces of globalisation, enabling circular migration by young doctors by including periods of training abroad in structured training programmes. This fulfils their desire to experience living and working in other cultures, as well as acquiring skills that source countries need. Ireland’s International Medical Graduate Training Initiative provides medical graduates from Pakistan and Sudan with a time-limited (two year) bespoke postgraduate training experience in Ireland, after which they must return to their home country for the training to be certified.^4^ The initiative is a direct response to Ireland’s failure to provide the training needed by most of the doctors that it recruits internationally, as required by the Global Code on the International Recruitment of Health Personnel.^1,12^

 However, inflow and outflow measures and relocation incentives have limited usefulness when confronted with the simple market forces of supply and demand, due to the growing global shortage of health professionals.^13^ Radical restructuring and growth in national and thereby the global health workforce are required. Arnold’s ‘thought exercise’ on expanding medical education would have been a non-starter in Ireland up to 2020.^14^ This is due to the limited number of hospital clinical placements needed to cater for the annual 500 non-EU medical students, who subsidise Ireland’s under-funded medical schools, as well as placements for the 725 EU nationals who are meant to form Ireland’s future medical workforce.^7^ However, the necessity of delivering remote learning during the COVID-19 pandemic and increasingly sophisticated simulation learning facilities are changing the medical education landscape. If countries increase their production of doctors, this may reveal – and reveal the need to tackle – national medical monopolies that benefit from shortages of doctors. Moreover, research shows that tomorrow’s hospital consultants and general practitioners are looking for work-life balances that might allow for trade-offs between income and a better work: life balance, requiring and enabling the employment of more doctors to staff national health services.^15^

 Too often, LMICs are seen as the victims of doctor migration and too seldom are they seen as the sources of solutions to the global health workforce crisis. Task-shifting, as proposed by Arnold,^14^ has been shown to be a feasible, effective and cost-effective way of delivering high quality essential surgical care at district hospitals in Africa.^16-18^ The development of alternative clinical cadres and adaptation of proven task-shifting strategies to high-income countries will require a culture shift in communities that will need to learn to trust and value quality-assured skills, more than qualifications. It will also require a shift towards more partnership models for medical doctors, from whom standardised routine care is shifted, who will need to learn to value and work with non-medical clinical colleagues.^19^ For Ireland’s exhausted doctors, this would not be a big ask. Profound shifts in where and how patients access care will also come from the changes imposed by the COVID-19 pandemic, where access to and consultations with general practitioners and hospital specialists are now increasingly mediated through remote video-platforms such as zoom.^20^

## COVID-19 – Impact and Opportunity

 So, how has COVID-19 impacted on Irish medical workforce numbers? At first sight, there appear to be positives: from 2019 to 2020, total NCHD numbers increased by 10.0%. Most were trainees, whose numbers increased by 14.9% compared with a 1.8% increase in non-trainees.^7^ However, over 40% of the increase in trainees (261/629) were interns, ie, medical graduates undertaking their first, pre-registration year of training; and “This increase is for one year only, as a direct result of the COVID-19 pandemic.”^7^ Humphries et al report little change in 2020 in the factors that were pushing Irish doctors to leave, from the perspective of NCHDs who returned to Ireland to support the COVID-19 response, and that of other NCHDs who were contemplating leaving.^21^ The authors state that “the pandemic intensified and reinforced, rather than radically altered, the dynamics of doctor emigration from Ireland.” Between 2019 and 2020, there was no change in the 4% of consultants in unapproved posts, nor in the 6%-7% of working consultants not registered on the specialist division register of the Medical Council.^7^ Most are consultants who have not completed specialist training, almost all of whom work in smaller hospitals, raising serious questions about the quality of care provided to rural populations.^3^ Tankwanchi et al are right to situate Irish doctor emigration in the context of the experiences of the international doctors that Ireland recruits to work in posts that Irish trained doctor have long rejected.^22^ As they may surmise, international (foreign-trained) doctors have similar and often worse experiences than their Irish counterparts in terms of poor working conditions and lack of training opportunities, as demonstrated in our earlier research.^23,24^

 Some green shoots are appearing in the wake of the COVID-19 pandemic, although these may yet turn out to be clutched-straws, such as a new public health medicine specialist contract after decades of second-class treatment of the specialty.^25^ This suggests recognition of the leading role public health professionals have played in Ireland, and globally, in the COVID-19 response. Doctors, nurses and other frontline health workers in every country have been the heroes of 2020 and 2021; and the importance of health systems and health workers to the survival of human society has never been clearer. Amri and Drummond have drawn on Baumgartner and Jones’ punctuated equilibrium theory of how public policy is developed, to explain how a shock such as COVID-19 can bring about major changes to policy after long periods of stasis.^26^ One of the obstacles to unblocking the logjam to doctor retention in Ireland has been the perception that hospital consultants are overpaid – a perhaps justified perception in respect to those who have lucrative private practices alongside their public sector contracts – which has made it difficult to justify the required investment in making consultant posts more attractive. Although the inequity of a two-tier consultant contract has been a bigger deterrent than the salary level to consultant recruitment^4^. More important still are the poor and sometimes intolerable working conditions for hospital consultants that have deterred Irish trainees from taking up consultant positions in Ireland.^1,4^ Frustration with public sector practice is a reason why some specialists opt for private practice.

 Until the COVID-19 pandemic struck Ireland, the media had a more ambivalent attitude to doctors than to nurses, who are invariably portrayed as frontline heroes. Now may be the opportunity to reframe media and public perceptions of the medical profession, changing the ‘policy image,’ which Baumgartner and Jones, as reported by Amri and Drummond,^26^ define as “how public policies are discussed in public and in the media.” The radical health systems and workforce changes that are required go far beyond renegotiating the terms and conditions of consultants’ contracts. Ireland’s 10-year national health service plan envisages restructuring the health system towards an integrated, primary care driven model, changing the roles and relationships of health professionals and creating more attractive career paths for Irish medical graduates.^8^ The COVID-19 shock has resulted in a policy environment that is now favourable for the Government to commit the level of investment needed to reconfigure the national health workforces to meet the future challenges and the pandemics that will follow.

## Ethical issues

 Not applicable.

## Competing interests

 Author declares that he has no competing interests.

## Author’s contribution

 RB is the single author of the paper.
